# Important parameters should be paid attention in PTMC radiofrequency ablation

**DOI:** 10.1038/s41598-023-40532-8

**Published:** 2023-08-18

**Authors:** Yiping Dong, Yanling Cheng, Peile Jin, Jifan Chen, Sohaib Ezzi, Yajun Chen, Jianing Zhu, Yanan Zhao, Ying Zhang, Zhiyan Luo, Yurong Hong, Chao Zhang, Pintong Huang

**Affiliations:** 1 Department of Ultrasound in Medicine, The Second Affiliated Hospital, Zhejiang University School of Medicine, Hangzhou, 310009 People’s Republic of China; 2 Department of Nursing, Xijing 986 Hospital, Air Force Medical University, Xi’an, 710054 People’s Republic of China; 3 Zhejiang University School of Medicine, Hangzhou, 310058 People’s Republic of China; 4 Research Center for Life Science and Human Health, Binjiang Institute of Zhejiang University, Hangzhou, 310053 People’s Republic of China

**Keywords:** Health care, Risk factors

## Abstract

In order to provide clinical references for the RFA procedure and to study the pivotal factors affecting the recovery time of radiofrequency ablation (RFA) in patients with papillary thyroid microcarcinoma (PTMC), 176 patients with low-risk intrathyroidal PTMC were included in this research. We randomly divided the whole cohort into training and test groups at a ratio of 7:3. The two-sample t-test was used to detect differences between the two groups. Least absolute shrinkage and selection operator (LASSO) regression was used to select the best predictor variables for predicting the status of RFA zone. Multiple test methods were used to ensure the scientific nature and accuracy of the Cox proportional hazards model. We tested the performance for the parameters and revealed the best cut-off value of each variable by the ROC curve and log-rank tests. The results showed patients aged above 49 years old, with RFA energy above 2800 J, the average diameter of the original tumour above 0.6 cm, or the average diameter of ablation zone at 1 month after RFA above 1.1 cm are risk factors for RFA zone delayed healing.

## Introduction

Papillary thyroid microcarcinoma (PTMC) is a form of papillary thyroid cancer (PTC) with a maximum diameter of 1 cm^1^. Due to its tiny size, PTMC is mainly detected by ultrasound examination^2^. In recent years, with the promotion of ultrasonography and fine-needle aspiration biopsy, PTMC has become the most common endocrine malignancy worldwide, and the morbidity rate has increased significantly^3^. Although PTMC is almost asymptomatic, well-differentiated, less invasive, unlikely to spread regionally^4^, and has a good prognosis^5^. Surgery, which usually resects the lesion lobe of the thyroid gland and regional lymph nodes, remains the most common treatment for thyroid carcinoma^6^. However, many studies, include ours, have shown that radiofrequency ablation (RFA) can be an effective treatment option for PTMC, with long-term follow-up studies reporting low rates of local recurrence and excellent tumor control^7,8^. In addition, regular monitoring and surveillance are necessary to detect any potential recurrence or progression of the disease^9^.Therefore, the need for substantial radical resection in certain individuals is being questioned due to the danger of surgical complications and a reduction in postoperative quality of life. These sparked the idea of customized PTMC treatment, which centred on RFA.

**Figure 1:**
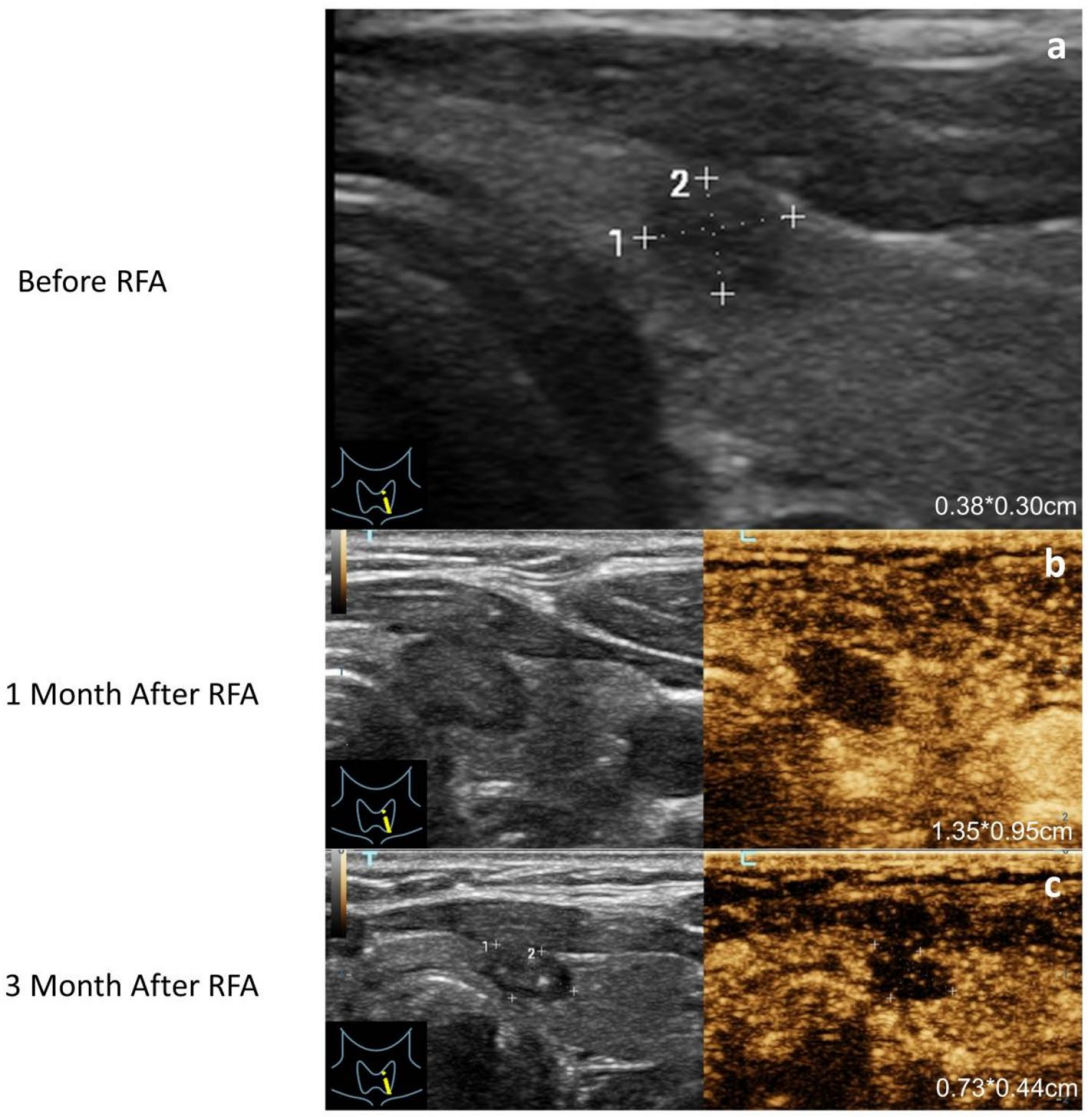
Ultrasound images of PTMC locoregional lymph nodes status in different treatment stages. (**a)** The original image of PTMC in two-dimensional ultrasound (2D-US). (**b**) 2D-US and CEUS images of 1 month after PTMC RFA. (**c**) 2D-US and CEUS images of 3 months after PTMC RFA.

As an ultrasound-guided interventional technique, RFA is a less invasive alternative to surgery^10^. RFA was first utilized to treat supraventricular tachycardias and depended on the creation of high-frequency alternating electromagnetic energy that caused thermal damage to the target lesions^11^. In recent years, ultrasound-guided RFA has been successfully conducted in PTMC, and it has been proven to be both safe and effective^12^. By means of thorough preoperative counseling, assessment, and anesthesia, the specialist physician inserts electrodes into the target area, utilizing thermal effects to achieve the therapeutic effect of ablating PTMC. The postoperative evaluation of the therapeutic effectiveness of RFA is performed through follow-up using contrast-enhanced ultrasound (CEUS) (Fig. 1). CEUS examination indicating no contrast agent enhancement in the ablation target area serves as an indicator of surgical success.

The 2017 RFA guideline of the Korean Society of Thyroid Radiology acknowledged RFA as a therapeutic option for patients with primary thyroid cancer who refuse surgery or who are unable to undergo surgery^10,13^. Research findings from scholars in Asia, Europe, the Americas, and other regions consistently suggest that RFA is likely to be superior to first-line surgical treatment options in the management of PTMC. Moreover, RFA demonstrates favorable performance in terms of intraoperative and postoperative complication control, as well as 5-year disease-free survival rates^7,9,14^.

Despite the widespread acceptance of RFA among physicians and patients, the standardization of the procedure remains relatively empirical. There exist variations in the energy and time parameters employed for ablating PTMC, underscoring the need for quantification and improved precision^15^. As a result, further research is imperative to mitigate potential risks associated with RFA and establish standardized guidelines. Patient management following RFA involves a range of tailored approaches specific to individual cases. During follow-up monitoring, the majority of patients demonstrate a gradual reduction in the size of the RFA zone, indicating successful treatment. However, it is important to note that a small percentage of patients may experience recurrence or incomplete ablation, necessitating alternative measures such as open surgery (e.g., thyroidectomy) for intervention. Additionally, in some cases, despite the absence of evidence of disease progression, the RFA zone may exhibit a lack of further shrinkage and persist as an unperfused small area. These observations highlight the complexities and nuances associated with RFA outcomes and the importance of ongoing research in addressing patient’s concerns.

Many postoperative follow-up patients are deeply concerned about the complete absorption time of the RFA zone. To provide more accurate answers to patients, we reviewed our previous clinical work and research findings^8^. Among the cases that were followed up for 5 years without any evidence of disease progression or recurrence, a portion of patients exhibited complete absorption of the RFA zone within a few months after the procedure. On the other hand, in some patients, despite the absence of disease progression or recurrence even after several years of follow-up, the ablation zone remained incompletely absorbed. This indicates that the absorption time of the RFA zone varies among different patients, even in cases where the lesion is completely ablated. To explore the potential reasons for this difference and address patient concerns, we collected relevant clinical data in an attempt to identify parameters that significantly influence the absorption time of the RFA zone. Additionally, our goal is to develop a nomogram that provides guidance for clinical RFA procedures. We hope that this research will benefit patients and contribute to the advancement of RFA.

## Results

### Patient cohort baseline

**Figure 2:**
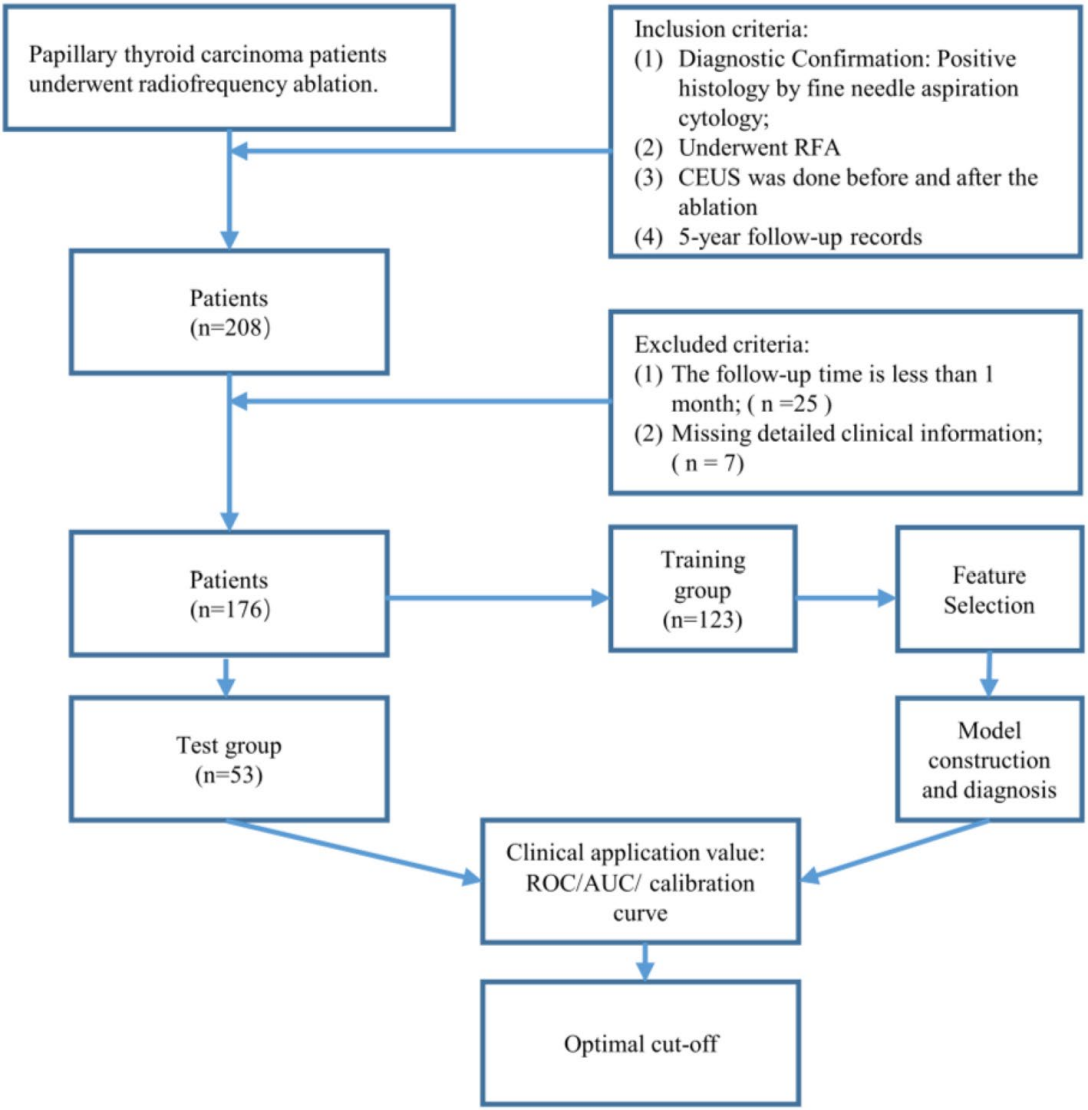
Flowchart of the patient recruitment pathway. RFA, radiofrequency ablation; CEUS, contrast-enhanced ultrasonography.

In this study, we obtained the medical records of patients with low-risk intrathyroidal PTMC from the Second Affiliated Hospital Zhejiang University School of Medicine. There were 208 patients meeting the above inclusion criteria and were all pathologically diagnosed with PTMC by fine-needle aspiration samples and underwent RFA. There were 32 patients without a proper follow-up ultrasound examination or enough clinical information were excluded. A total of 176 patients finally included in this study were randomly divided into training and test groups at a ratio of 7:3. The participants selection process is depicted in Fig. 2. The demographic characteristics of the patients in the training and test cohorts are summarized in Table 1. The average RFA energy was 3333.15 J (power range, 20–40 W). No local recurrence or distant metastases were detected during the 5-year follow-up period. There are no significant differences between the two groups (*P* < 0.05).

**Table 1:** Baseline characteristics of patients with low-risk intrathyroidal PTMC from The Second Affiliated Hospital Zhejiang University School of Medicine.

	All patients	Training cohort	Test cohort	*P-*value
Participants	176	123	53	
Age (years)	41.17	41.08	41.38	0.877
Energy(J)	3333.15	3386.69	3208.92	0.805
The mean diameter of original tumour (cm)	0.56	0.57	0.55	0.827
The mean diameter of RFA zone				
1 day after RFA (cm)	1.95	2.17	1.42	0.355
1 month after RFA (cm)	1.11	1.1	1.14	0.534
Sex				0.273
Male	36	27	9	
Female	140	96	44	

### LASSO regression and feature selection

**Figure 3:**
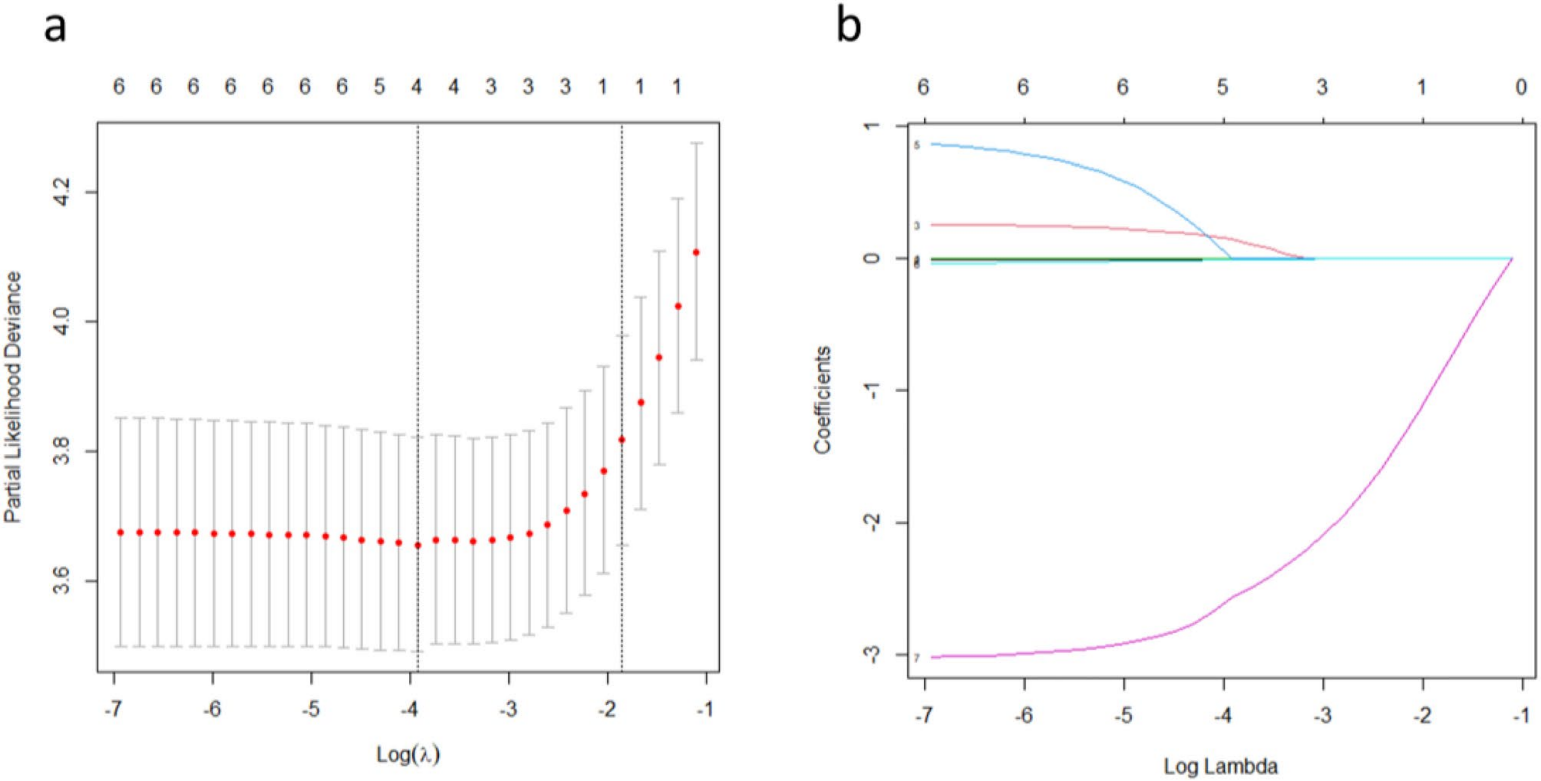
The prognostic factors of RFA focus absorption time were selected by the least absolute shrinkage and selection operator (LASSO) regression model. (**a**) The optimal values of the LASSO tuning parameter (*λ*) are indicated by the dotted vertical lines. (**b**) LASSO coefficient profiles of the 6 candidate features.

Least absolute shrinkage and selection operator (LASSO) regression analysis prevents overfitting and has very good performance in selecting variables^16^. We used LASSO regression analysis to select prognostic factors in the patient training cohort. The *z* values for each factor were as follows: sex (− 1.935), age (0.856), RFA energy (− 0.812), the average diameter of original tumour (1.659), the average diameter of ablation zone at 1 day after RFA (− 0.442), and the average diameter of ablation zone at at 1 month after RFA (− 6.350). The vertical dashed lines in Fig. 3a represent the optimal value variables. The lower horizontal axis in Fig. 3b represents the magnitude of the lambda value in the LASSO regression model. The upper horizontal axis represents the number of variables in the model whose coefficients are not zero at the time. Three prognostic variables, age, the average diameter of original tumour, and the average diameter of RFA zone at 1 month after ablation, were selected by LASSO regression analysis using the minimum standard value as the criterion. Since the RFA energy had a significant relationship with the ablation zone^17^, experts suggested it should also be considered a candidate factor. Thus, four prognostic variables were used to build the foci absorption prediction model.

### Construction and diagnosis of absorption time nomogram

**Figure 4:**
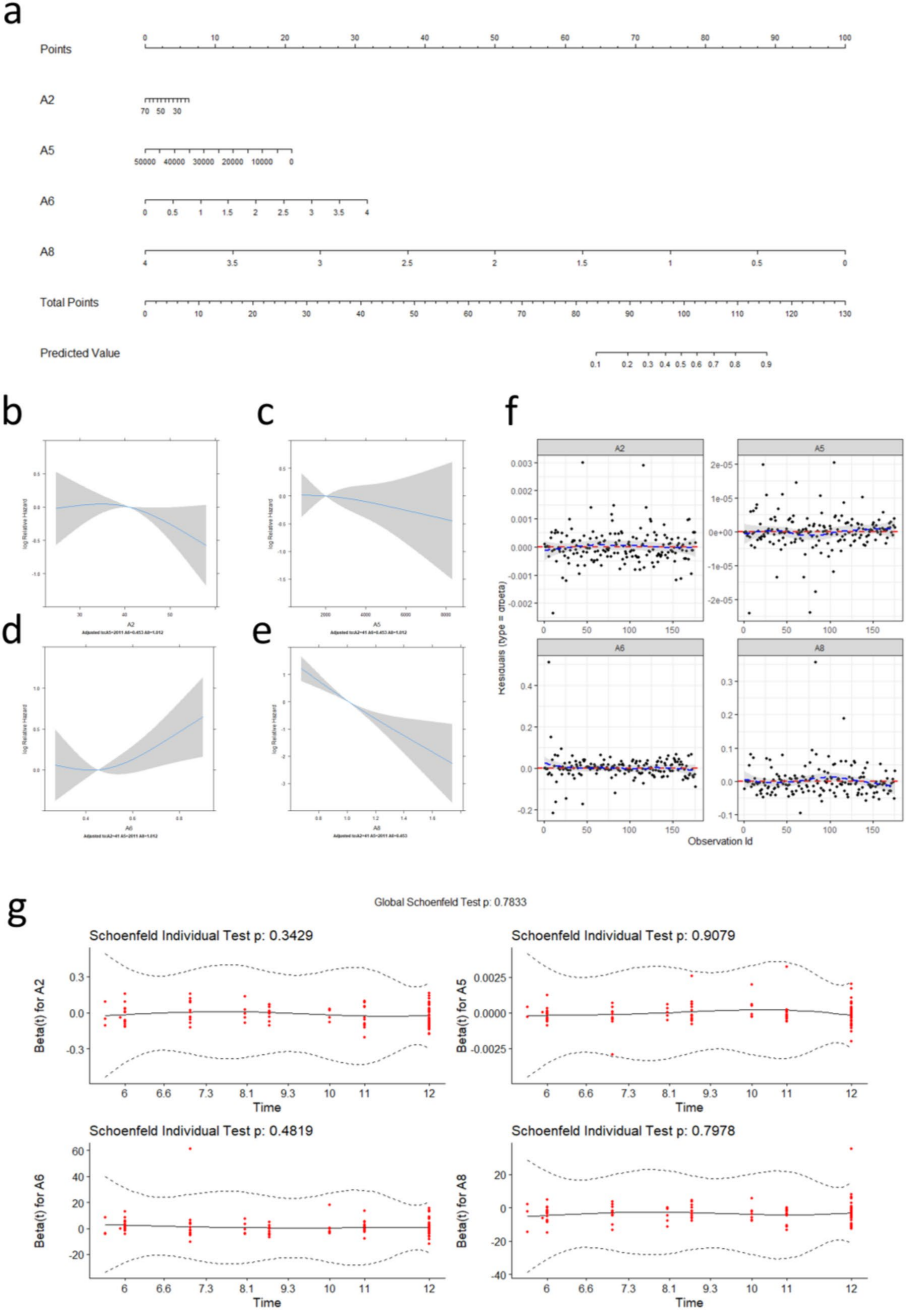
The construction and diagnosis t-test of the absorption time nomogram. (**a**) The nomogram, combining the patient’s age (A2), the average diameter of original tumour (A6), the average diameter of RFA zone at 1 month after ablation (A8), and RFA energy (A5), developed in the training set. (**b–g**) The model was diagnosed by a linear relation test (Fig. 3b–e), influential case test (Fig. 3f), and proportional hazards assumption test (Fig. 3g).

By using the aforementioned four variables, we constructed the PTMC RFA absorption model by Cox regression, and a nomogram prediction model is shown in Fig. 4a. Then, we diagnosed the model by a linear relation test (Fig. 4b–e), influential case test (Fig. 4f), and proportional hazards assumption test (Fig. 4g). Next, we conducted a multicollinearity test. The vif value of each variable was age (vif = 1.015), RFA energy (vif = 1.343), the average diameter of original tumour (vif = 1.286), and the average diameter of RFA zone at 1 month after the procedure(vif = 1.381). These data reflected that the building process of this model was scientific and rigorous.

### Accuracy of the nomogram

**Figure 5:**
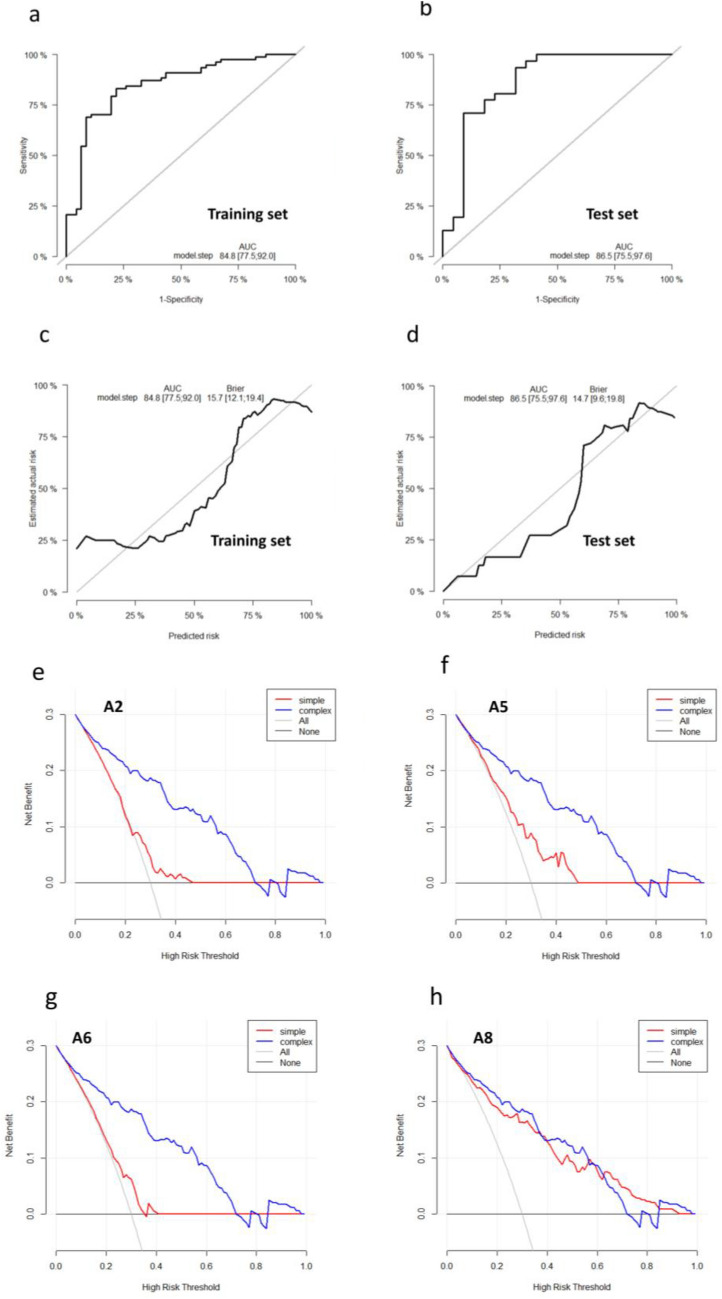
Test of the accuracy of the nomogram. (**a, b**) Evaluate the accuracy of the nomogram by ROC curve and AUC values in the training and test groups. (**c, d**) Calibration curves for the nomogram in each cohort. (**e–h)** DCA decision curve analysis for each prognostic variables: patient’s age (A2), RFA energy (A5), the average diameter of original tumour (A6), and the average diameter of RFA zone at 1 month after ablation (A8).

To evaluate the accuracy of the nomogram, we used the “Survival ROC” package to draw the ROC curve of the training (Fig. 5a) and test cohorts (Fig. 5b). The AUC values of the two cohorts are shown in the figures, which were 0.848 and 0.865, respectively. Meanwhile, the training and test cohort calibration plots (Fig. 5c,d) exhibited good agreement between the predictions and actual observations. The Brier scores were 0.157 and 0.147, respectively (Fig. 5c,d). These data indicate that the accuracy of the constructed model is reliable.

### Decision curve analysis

Decision curve analysis (DCA) was used to evaluate the clinical net benefit of the predictive model^18^. In Fig. 5e–h, the abscissa is the threshold probability, and the ordinate is the net gain rate. Our findings suggest that the nomogram model is effective in predicting the RFA zone absorption time of patients with PTMC (Fig. 5e–h).

### RFA zone absorption time analysis

**Figure 6:**
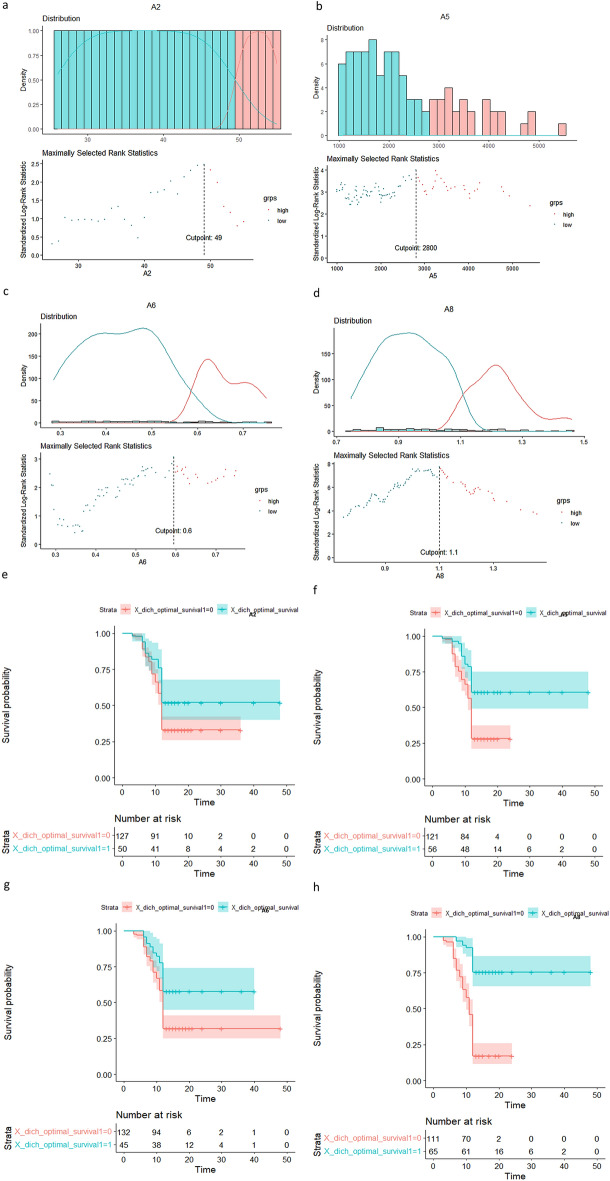
The optimal cut-off points for each selected prognostic factor (**a–d**) and the RFA focus healing probability within 12 months of the whole patient cohort in the optimal cut-off points (**e–h**).

In our data, the patient’s RFA zone absorption time ranged from 3 to 48 months, with an average absorption time of 12.8 months. We defined that patients whose RFA absorption time was greater than 12 months as the “high-risk group”, and the rest were defined as the “low-risk group”. Nextly, we applied the log-rank test to find the optimal cut-off points for each selected prognostic factor. The calculation results are displayed in Fig. 6a–d. Therefore, from our results, the main factors affecting the RFA zone absorbtion were age greater than 49 years old, RFA energy greater than 2800 J, and the average diameter of ablation zone greater than 1.1 cm at 1 month after RFA (Fig. 6a–d). Figure 6e–h shows the RFA zone healing probability within 12 months of the whole patient cohort in the optimal cut-off points for each selected prognostic factor.

## Discussion

Many studies have shown that RFA has a good therapeutic effect in patients with PTMC. However, the description of the RFA treatment process in patients with PTMC in clinical guidelines is relatively empirical. The RFA power used for PTMC patients are varied from several watts to dozens watts among different medical institutions, so did the RFA time, for example. Although a large number of studies have shown that RFA can significantly benefit PTMC patients in terms of efficacy and quality of life, more energy is not always better in RFA surgery^14^. Therefore, there are many factors that we need to quantify and clarify. To address patient’s concerns, based on our clinical RFA data, by using LASSO regression and Cox regression, we selected four key factors and developed a clinical prediction model. The key signatures can well distinguish whether the absorption time of RFA zone in PTMC patients is greater than 1 year. Incorporating the clinical risk factors into an easy-to-use nomogram facilitates the doctors referring to before surgery and facilitates the individualized prediction of RFA zone absorption time after surgery.

PTMC is the most common endocrine malignancy worldwide, and its morbidity rate has increased significantly. The disease is usually asymptomatic, well-differentiated, less invasive, and unlikely to spread regionally. Although surgery remains the most common treatment for PTMC, many influential studies have shown that ultrasound-guided RFA is highly effective and safe for treating PTMC^19,20^. Patients with PTMC who decline surgery or active surveillance can benefit from RFA as a minimally invasive therapy^21^. RFA is not oncologically inferior to open surgery for carefully selected patients with low-risk intrathyroidal PTMC, and it is associated with a superior quality of life, lower complication rate, and reduced overall expenses^22^. Although RFA is a comparatively new technology, many studies and practiceshave shown that RFA is an alternative to surgery in patients with low-risk intrathyroidal PTMC^23^. However, the guidelines for the use of RFA are relatively brief and lack details, and doctors around the world need a clear consensus on the specific operation of RFA.

By assessing the predictor–outcome correlation and decreasing the regression coefficients with the LASSO approach, six prospective clinical features were reduced to four viable predictors for the prediction model. This strategy outperformed the method of selecting predictors based on the strength of their univariable connection with the outcome, allowing a nomogram to be constructed from a panel of selected characteristics. Recent studies have incorporated multimarker analyses that combine individual markers into marker panels. Such as RNA sequencing and the clinical data of patients with gastric cancer revealed that the interaction of m6A alterations and lncRNAs may play a key role in predicting gastric cancer prognosis, shaping the tumour immune milieu, and predicting immune checkpoint inhibitor therapeutic responses^24^. Similarly, the panel of selected features that combine multiple individual imaging features can be better applied to predict KRAS mutation status in colorectal cancer^25^.

Given that the RFA zone recovery time was comparable in the patient cohorts, the improved discrimination implies that the complex signature was robust for prediction and could be applied directly in the test cohort. In this study, we used our data to build a clinical model and provide a reference for clinical practice. The data of 176 patients with low-risk intrathyroidal PTMC were collected and randomly divided into the training and test groups. No instances of neck hematoma, skin burns, dysphonia, local infection, or injury to vital neck organs were observed in the whole corhort. Using LASSO regression analysis and Cox regression, we built a clinical model that can predict foci absorption time after RFA. By applying the log-rank test, we noted that the age of the patient, RFA energy, the average diameter of original tumour, and the average diameter of RFA zone at 1 month after ablation were prognostic variables for the RFA zone absorption time. In addition, the patient’s age above 49 years old, an ablation energy above 2800 J, an average diameter of original tumour above 0.6 cm, or the the average diameter of RFA zone above 1.1 cm at 1 month after ablation are risk factors for ablation foci healing within 12 months. These findings tell us that when a patient has the aforementioned risk factors, his/her expected RFA absorption time should be prolonged. In particular, we should note that there may be the best cut-off points for total energy during RFA in patients with low-risk intrathyroidal PTMC.

The energy used in RFA surgery is very important. The RFA range need to be greater than the tumour edge to prevent marginal residue and recurrence. When all portions of the target ablation area had changed to transient hyperechoic zones, the ablation was terminated^25^. From the published reports, the RFA power varies from several watts to dozens of watts, and the ablation time also varies according to the doctor’s experience. If the RFA energy is insufficient, the foci may not be eliminated. If too much energy is used to achieve a larger ablation area, it may lead to excessive ablation and even normal tissue damage in the patient. This attribute bears some resemblance to tumor radiotherapy. Therefore, according to our data and model, we drew a line of the total RFA energy at 2800 J to prevent overablation. Despite the widespread popularity of RFA globally, particularly in Europe and Asia, this study maybe represents a pioneering effort in investigating the optimal RFA energy dosage for patients with PTMC. We hope this research can benefit patients and contribute to the development of RFA.

Study limitations include the fact that the nomogram was developed using retrospectively collected data from a cohort of patients at a single centre, and the sample size of the study was relatively small. Therefore, confounding bias, admission bias (Berkson bias), current case-new case bias (Nyman shift) are unavoidable in this study. Further research is needed to validate the performance of the nomogram in a larger external validation cohort.

Taken together, our results highlight the vital factors that influence RFA zone absorption time. As a retrospective study, we developed a nomogram based on the clinical data. It can help doctors in risk assessment of patients and in careful planning of RFA procedure. The result of this study may provide a new view of RFA procedure.

## Methods

### Study population

This retrospective study was approved by the Institutional Review Board (IRB) of the Second Affiliated Hospital Zhejiang University School of Medicine (SAHZU). The requirement for the informed consent from the patients was waived by the IRB of SAHZU, and all experiments were performed in accordance with relevant guidelines and regulations. The inclusion criteria were as follows: (1) patients with a definitive biopsy-proven pathological diagnosis of PTMC; (2) patients who underwent RFA of thyroid lesions, without extrathy roidal invasion and no lymph node metastasis in former imaging studies; (3) patients who had undergone CEUS examinations before and after radiofrequency ablation; and (4) patients with complete 5-year clinical data. The exclusion criteria were as follows: (1) patients with a follow-up time less than 1 month or (2) patients with missing detailed case information. A total of 176 patients with cytopathologically confirmed PTMC were enrolled in the study between July 2015 and July 2017.Among the patients who underwent RFA surgery between 2015 and 2017, there were no cases of dysphagia, permanent hoarseness, hematoma, lymphatic cysts, chylous leakage, or neurological complications. One patient experienced temporary hypoparathyroidism, while two other patients had transient voice changes. Among the cases included in the study cohort based on the aforementioned inclusion and exclusion criteria, no adverse intraoperative or postoperative reactions were observed. The patient recruitment pathway is presented in Fig. 1. Then, the patient cohort was divided into two independent sets: 123 (70%) patients were randomly selected for the training group, and 53 (30%) patients were randomly selected for the test group. The clinical characteristics of patients, including sex and age, etc., were derived from clinical records.

### Protocol of CEUS

CEUS examinations were conducted utilizing a Resona 7 machine (Mindray Company, China) equipped with UWN^+^ technology. Each patient underwent scanning with an L 11–3 WU 80 (3–11 MHz) linear array transducer. The mechanical index (MI = 0.05–0.10) was automatically determined by the system based on the beam-focus depth. The contrast agent used in this study was SonoVue (Bracco SpA, Milan, Italy), supplied as a lyophilized powder. The reconstitution process involved adding 5 mL of 0.9% saline to the vial and gently shaking it by hand to achieve a homogeneous microbubble suspension. For contrast enhancement, a 19-gauge cannula was skillfully inserted into an antecubital fossa vein. Subsequently, 1.2 mL of SonoVue was administered as a bolus, immediately followed by a 5 mL saline flush through a three-way tap for each contrast study. The entire movie sequence, lasting at least 2 min, was meticulously preserved for subsequent analysis. During postoperative follow-up, examinations were conducted at 1, 3, and 6 months using neck ultrasound and CEUS to assess tumor recurrence and cervical lymph node status. The enhancement pattern of the ablation zone was evaluated on CEUS examination based on the following criteria: (a) complete ablation, where the entire ablation area exhibited non-enhancement; (b) recurrence or incomplete ablation, characterized by enhancement in any part of the ablation zone. Abnormal findings indicative of cervical lymph node metastasis included a globular shape, loss of the normal echogenic hilum, peripheral flow instead of hilar flow, heterogeneity with cystic components, and presence of microcalcifications. Suspicious lymph nodes were subject to biopsy.

### Protocol of RFA

Radiofrequency energy was generated using a VIVA radiofrequency generator (STARmed, Goyang, Korea) and an 18-gauge monopolar internally cooled electrode (VIVA; STARmed). Continuous cooling with distilled water circulation prevented overheating of the electrode shaft. The patient was positioned supine with a slightly extended neck. Local anesthesia with 1% lidocaine was administered, and fluid was injected between the tumor and surrounding tissues to create a distance from the tumor, thereby mitigating the risk of heat-related injury. Ablation was performed under ultrasound guidance with an energy output of 20–40 W until complete tumor coverage. Immediately following the ablation, CEUS was conducted to assess the ablation zone. If residual tumor tissue was suspected based on CEUS findings, repeat RFA was performed to achieve comprehensive tumor eradication. Neck compression was applied for a duration of 30 min. The duration of the procedure, as well as any intraoperative complications, were diligently documented. Following the ablation, each patient remained under observation at the hospital for a period of 1–2 h. Postoperative follow-up examinations were conducted at 1, 3, and 6 months using neck ultrasound and CEUS. Subsequently, every 6 months, the ablation zone and tumor recurrence were evaluated using two dimensional ultrasounds (2D-US). At 3 months post-RFA, an ultrasound-guided fine needle aspiration biopsy (FNAB) would performed on the RFA zone to confirm the therapeutic efficacy.

### US image acquisition

Before RFA, the ultrasound features of PTMC typically included a hypoechoic nodule with well-defined margins, a taller-than-wide shape, and intranodular vascularity on Doppler ultrasound. After RFA, the ultrasound features of the treated area would vary based on the degree of ablation. Ideally, the treated area should show non-enhancement in the entire ablation zone, indicating complete ablation. However, in cases of incomplete ablation or tumor recurrence, there may be areas of enhancement within the ablation zone. It is important to monitor these ultrasound features during follow-up to evaluate the effectiveness of the RFA treatment^26^. The ultrasonography results were collected by patients’ ID. Patients included in our study underwent both 2D-US and CEUS examinations before and after RFA. The tumor and RFA zone were appropriately described in the imaging results.

### Construction of the clinical predictive model

We collected the following clinical data of the cohorts as potential variables for the predictive model: patient age, sex, the energy of RFA, the average diameter of the original tumour, and RFA range at different time points (1 day after RFA and 1 month after RFA). In addition, the RFA focus absorbtion time of each patient were also documented and gathered as endpoint of our research. Based on these clinical data from The Second Affiliated Hospital Zhejiang University School of Medicine, the training set of our predictive model had a size of 123 people, and the test set had a size of 53 people. The construction of the model was based on R software.

### Statistical analysis

All statistical analyses were performed using R software 4.1.1 (“https://www.r-project.org”). A total of 176 patients were randomly divided into a training dataset and a test dataset at a ratio of 7:3. We used a two-sample t test to detect differences between the two datasets. Then, LASSO regression was used to select the best predictor variables for predicting the status of the RFA foci. All the included variables underwent the linear relation test, influential case test, proportional hazards assumption test, and multicollinearity test, which ensured the scientific nature and accuracy of our model. A Cox proportional hazards model was used to construct the prediction model. A nomogram was used to intuitively predict the status of the RFA foci. The ROC curve was used to compare the diagnostic performance and suggest the best cut-off value of each predictor variable. Log-rank tests were used to compare differences between the high-risk and low-risk groups. All test results with *P* values less than 0.05 were considered statistically significant.

## Data Availability

The datasets used and/or analysed during the current study available from the corresponding author on reasonable request.
